# Neural Substrates of Homing Pigeon Spatial Navigation: Results From Electrophysiology Studies

**DOI:** 10.3389/fpsyg.2022.867939

**Published:** 2022-04-06

**Authors:** Gerald E. Hough

**Affiliations:** ^1^Department of Biological Sciences, Rowan University, Glassboro, NJ, United States; ^2^Department of Psychology, Rowan University, Glassboro, NJ, United States

**Keywords:** hippocampus, adaptive evolution, spatial memory, birds, action potentials, space perception

## Abstract

Over many centuries, the homing pigeon has been selectively bred for returning home from a distant location. As a result of this strong selective pressure, homing pigeons have developed an excellent spatial navigation system. This system passes through the hippocampal formation (HF), which shares many striking similarities to the mammalian hippocampus; there are a host of shared neuropeptides, interconnections, and its role in the storage and manipulation of spatial maps. There are some notable differences as well: there are unique connectivity patterns and spatial encoding strategies. This review summarizes the comparisons between the avian and mammalian hippocampal systems, and the responses of single neurons in several general categories: (1) location and place cells responding in specific areas, (2) path and goal cells responding between goal locations, (3) context-dependent cells that respond before or during a task, and (4) pattern, grid, and boundary cells that increase firing at stable intervals. Head-direction cells, responding to a specific compass direction, are found in mammals and other birds but not to date in pigeons. By studying an animal that evolved under significant adaptive pressure to quickly develop a complex and efficient spatial memory system, we may better understand the comparative neurology of neurospatial systems, and plot new and potentially fruitful avenues of comparative research in the future.

## Introduction

All vertebrates require a strong spatial memory system to store and retrieve important locations in their environment, so that they may navigate amongst them. Some species are better than others due to behavioral requirements like long-distance migration, scatter hoarding, or other complex behavioral needs. While there has been a half century of productive research investigating the neural systems of spatial memory in rodents and primates (e.g., O’Keefe and Dostrovsky, 1971; Moser et al., 2008), studies in non-mammalian vertebrates with significant spatial memory needs were far less common. Fortunately, avian studies are increasing in frequency and providing valuable comparative studies on the importance of the brain in coordinating spatial behaviors. In birds, which require good spatial memory to migrate and locate pertinent environmental cues, neural and genetic studies have been performed on black-capped chickadees (*Poecile atricapillus*, Pravosudov et al., 2012, 2013; Croston et al., 2015), homing pigeons (*Columba livia*, Bingman et al., 2003; Gagliardo et al., 2014; Herold et al., 2015), and a growing body of data collected in domestic chicks (*Gallus gallus*, Tommasi et al., 2012; Mayer et al., 2018; Morandi-Raikova et al., 2020). There are also studies in the tufted titmouse (*Baeolophus bicolor*, Payne et al., 2021), quail (*Coturnix japonica*, Ben-Yisahay et al., 2021), zebra finch (*Taeniopygia guttata*, Mayer et al., 2013), and the streaked shearwater (*Calonectris leucomelas*, Takahashi et al., 2022). In summary, all findings to date have supported the key role of the hippocampal formation in underlying spatial behaviors in birds.

**FIGURE 1:**
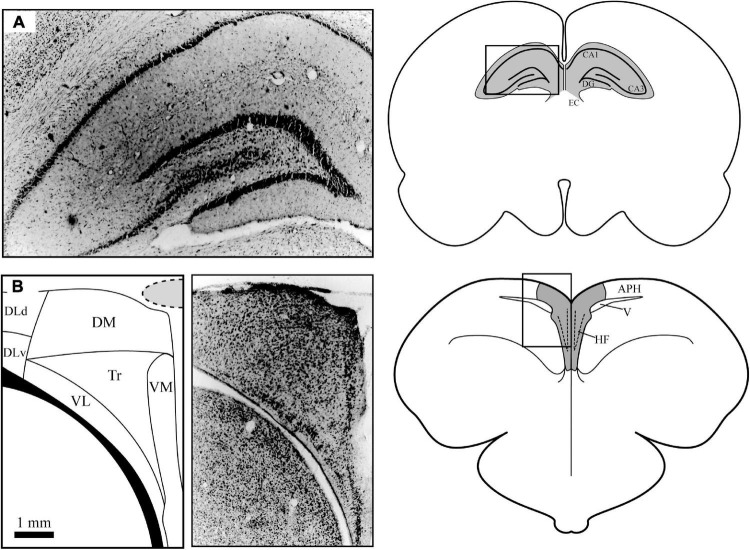
Anatomical differences between mammalian **(A)** and homing pigeon **(B)** brain anatomy. **(A)** The mammalian hippocampus (shaded) is located under a significant amount of neocortex (approximately 3 mm). The simplified flow of information through this structure is as follows: from entorhinal cortex (EC) to Ammon’s Horn CA3 to Ammon’s Horn CA1 to dentate gyrus (DG) and then back to EC. **(B)** The pigeon hippocampal formation (shaded) is a dorsomedial structure that overlies the lateral ventricle on the surface of the brain. The subdivisions in the avian HF include the following areas: dorsal and ventral dorsolateral (DLd and DLv respectively), dorsomedial (DM), ventrolateral and ventromedial dense cell layers (VL and VM, respectively), and the triangular subdivision (Tr). The area parahippocampal (APH) is not considered to be part of the avian HF but has substantial connections to it. The location of the overlying central sinus blood vessel is indicated by the shaded area. Photomicrographs of cresyl violet stained tissue were taken by the author. Pigeon subdivisions are based on Atoji and Wild (2006).

Both the hippocampus (Hp) of mammals (Figure 1A) and the hippocampal formation (HF) of birds (Figure 1B) play a key role in learning and memory; ablating this structure severely impairs spatial memory formation (Colombo et al., 2001; Bingman et al., 2003, 2005; Tommasi et al., 2003). Birds with higher spatial memory demands have larger relative hippocampal volumes compared to those that do not (Volman et al., 1997; Cnotka et al., 2008). How this structure processes aspects of the environment, and how it combines visual, magnetic, proprioceptive, and auditory inputs is still poorly understood, but a host of studies over the last two decades have attempted to understand how a brain structure so evolutionarily and morphologically dissimilar to the mammalian Hp has, under heavy selective pressure, turned a non-migratory and sedentary rock pigeon (Lowther and Johnston, 2020) into one of the premiere navigators of the animal world.

While not normally a species that has extensive spatial memory requirements, the rock pigeon has been selectively bred by humans to have exceptional navigational abilities, even at a young age. Over the last few millennia, humans have used members of this strain to return to a home loft from hundreds of miles away, even if the release point is from a location they have never visited (Blume, 2004). As a result of this strong adaptive selection, homing pigeons have developed a remarkable spatial memory system, one that rivals humans in their ability to quickly navigate across a three-dimensional environment using cues that are both time- and seasonally variable (Ioalè et al., 2000; Wiltschko et al., 2000). This paper will discuss some of the major findings made in understanding the neural substrates of homing pigeon spatial navigation.

### Avian vs. Mammalian Hippocampal Formation Analogies

While more extensive comparisons between the avian HF and mammalian Hp exist elsewhere (for instance, Puelles et al., 2007; Herold et al., 2015), a basic comparison is given here to put the neurophysiological investigations into context. The avian HF is a brain structure that has evolved independently in birds, with over 300 million years since there was a common ancestor between birds and mammals (Striedter and Charvet, 2008). Upon casual examination, there are striking visual differences between the avian and mammalian structures (Figure 1). The avian HF is located on the posterior dorsomedial surface of the brain, while the mammalian Hp is a more ventral, lateral structure. The avian HF, even in denser cell layers, is less dense than similar areas than the mammalian Hp. And the clear boundary between the mammalian Hp and surrounding structures is much less defined in the avian HF.

The subdivisions of the avian HF are also much less clearly defined, outside of the characteristic “V” of the dense ventral neuron layers seen in cresyl violet stained tissue. But over the years, scientists have used various anatomical, neurochemical, and tract tracing methods to develop various theories for how the HF is divided. Although still being debated, this review will use the subdivision nomenclature of Atoji and Wild (2006), which was developed in the homing pigeon and has the most relevance to the studies described in this review (Figure 1B). These subdivisions include a dorsolateral subdivision that has both dorsal and ventral sections (DLd and DLv), a dorsomedial subdivision (DM), and ventrolateral and ventromedial dense cell layers (VL and VM). Between the dense cell layers and under DM is a sparsely populated subdivision labeled ventrocentral (VC) in older literature but has been replaced by the label Triangular subdivision (Tr) in newer studies.

Despite the anatomical and developmental differences explained above, there do appear to be significant homologies between the avian and mammalian structures. Both the avian HF and the mammalian Hp are pallial in developmental origin (Reiner et al., 2004; Jarvis et al., 2005). There is also significant similarities in how the mammalian and homing pigeon hippocampal subdivisions communicate with each other chemically (summarized well in Atoji and Wild, 2006; Herold et al., 2015). In particular, neuropeptides expressed in distinct subdivisions of the mammalian Hp such as Substance P, neuropeptide Y, somatostatin, cholecystokinin, glutamate, and vasoactive intestinal polypeptide are also found in the avian HF (Erichsen et al., 1991; Krebs et al., 1991; Riters et al., 1999; Rosch et al., 2005; Rosinha et al., 2009). In newer studies, using a combination of tract-tracing and gene expression experiments, researchers have supported the idea that DM was homologous to Ammon’s Horn (CA3, CA1) and the dense cell layers VM and VL were homologous with dentate gyrus (Atoji et al., 2016). This does overlap with receptor-binding (Herold et al., 2014) and connectivity studies (Atoji and Wild, 2004; Atoji et al., 2018). Finally, recent gene expression has found functional similarities in coordinating spatial organization in chicks (Morandi-Raikova and Mayer, 2020, 2021).

The connections within the HF of homing pigeons are similar as well. In early studies, there appeared to be an feed-forward pathway through the HF that was remarkably similar to the trisynaptic pathway seen in mammalian Hp (Hough et al., 2002; Kahn et al., 2003). The avian pathway is not identical in its molecular components to mammals, as a later study demonstrated a lack of zinc staining in the avian HF that is characteristic of the mammalian Hp mossy fiber tract in pigeons (Tömböl et al., 2000). Studies have also suggested that information flows from dorsolateral to dorsomedial structures, passes through the dense ventral cell layers, then passes back out ventrolaterally as well as crosses over to the contralateral hippocampus (see Hough et al., 2002; Atoji and Wild, 2006). Later studies using more complex methods have found the connection pathways to be much more diverse (Atoji et al., 2016; Behroozi et al., 2017). The HF also receives input from a wide variety of sensory pathways, with particularly large inputs from visual centers like the Wulst (Atoji et al., 2018; Atoji and Wild, 2019), a crucial area for spatially mediated associative learning (Budzynski et al., 2002). Therefore, the exact patterns of information flow through the HF and connected brain areas, and its comparisons to the mammalian system, is still a matter of much academic debate but seem to strongly support the homology of the two structures.

There has been one key anatomical difference between mammalian and avian hippocampal structures, and that is how it changes across the lifespan. In older individuals in both groups, there is a progressive loss of working memory as individuals get older (Moss et al., 1988; Head et al., 1995; Rasmussen et al., 1996; Kukolja et al., 2009; Coppola et al., 2014a). But in contrast to mammals, which show declines in mammalian Hp neuroanatomy as individuals age (e.g., Bettio et al., 2017), the homing pigeon HF shows a significant increase in both size and neuronal density in older birds, but with less activity per neuron (Coppola et al., 2016; Coppola and Bingman, 2020). This increase in structural complexity at the cost of good spatial memory could be due to either runaway neurogenesis, which continues to incorporate a high number of immature HF neurons into adulthood (Kahn et al., 2001), or through decreased neuronal apoptosis to remove unnecessary neurons, where lower rates were seen in adult food-storing birds (Clayton and Krebs, 1994). A working hypothesis for this pattern is that the demand for excellent spatial working memory at an early age has increased the developmental speed of the HF that continues throughout the lifespan. Eventually, these runaway processes adversely affect spatial memory due to either disrupting pre-existing pathways or the inability to remove pathways to environmental cues that are no longer relevant (see Coppola et al., 2016 for more on this issue).

### Importance of the Hippocampal Formation in Spatial Behavior

Early lesion studies showed marked deficits in navigation following Hp ablation in a host of studies in the 1990s and beyond. The absence of a functional hippocampus causes a profound loss of navigational abilities (Bingman and Yates, 1992; Colombo et al., 2001; Bingman et al., 2005; Coppola et al., 2014b). In particular, the use of spatial cues is particularly impaired following HF damage (Coppola et al., 2014b). There are also marked learning vs. performance differences; impairments are seen when hippocampus is lesioned before learning, but not when maps were already acquired (Bingman et al., 1999; Gagliardo et al., 2004; Coppola et al., 2014b). In the zebra finch, researchers found evidence that the HF is active during retrieval as well, but the levels of gene activation were significantly less than during learning and there was no evidence of activation for a non-spatial task (Mayer et al., 2010). These results suggest that while it plays a role in retrieval, the avian HF is crucial for learning new spatial information.

While it is clear there are significant homologies in organization, connectivity, and biomarkers, our understanding of how HF neurons encode environmental space is restricted to a relatively small number of studies in the homing pigeon. Performing neurobehavioral experiments in these birds was a challenge that has required creative solutions. First, recording from the avian HF is more difficult due to its location in the brain. As explained previously, the HF is located on the posterior dorsomedial surface of the brain (Figure 1B), compared to the deeper and more lateralized position of the Hp in mammals (Figure 1A). Electrodes implanted into the dorsomedial hyperpallium run the risk of damaging the large central sinus blood vessel, which makes tetrode placement very difficult, and can damage the hippocampus due to lack of blood flow and increased mortality during the months of experimentation. The bifurcation of this large blood vessel around the cerebellum also makes penetrations into the posterior hippocampus extremely challenging. In addition, unlike the thick mammalian skull, the avian skull is thin and lattice-like that makes securing electrophysiological microdrives a challenge. As documented in other studies, the neural density of avian HF is much lower, which prevents researchers from recording from multiple isolated neurons simultaneously without using a very large array of microelectrodes. Finally, pigeons have large scale, bobbing head movements during walking that need to be eliminated; techniques that rely on rigid electrodes give significant artifacts, and any tethers need to be small and not impair movements or trigger fear responses in animals while being recorded in an experimental space.

**FIGURE 2:**
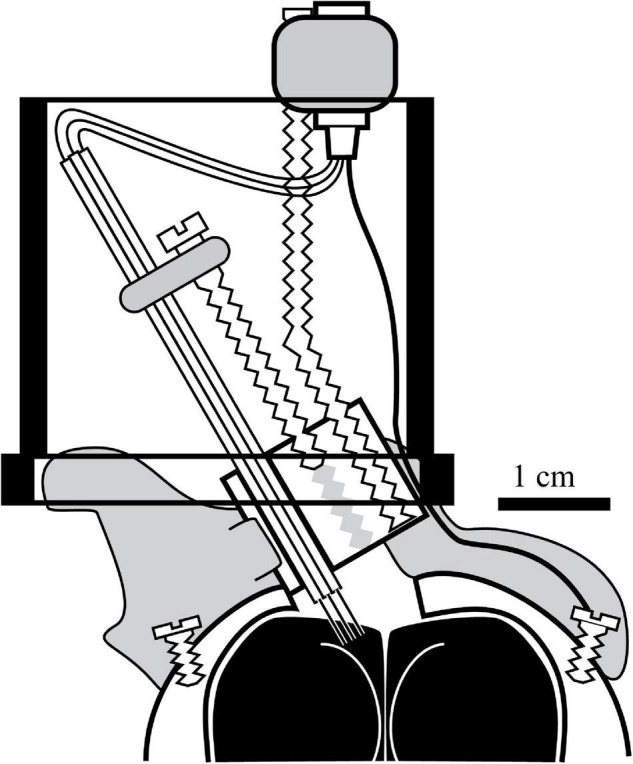
Headstage design used in the majority of the studies involving the Bingman lab. A custom Drive screws were tapped into a Delrin block and connected to bundles of tetrode microelectrodes such that every full turn of the drive screw drove the tetrode bundles 320-μm further into the brain. The wires were soldered into a 16-pot connector connected to the headstage *via* threaded rod that also had a ground connection that was secured to the brain *via* a separate wire. Once assembled, the headstage was secured to the skull using stainless steel screws that were covered with dental cement (gray shading), which also protected the small area of exposed brain. In two studies (Hough and Bingman, 2004, 2008), the drive was implanted at a 30-degree angle as pictured, to maximize coverage of the medial HF (dotted lines indicate representative tetrode trajectories) without damaging the central sinus on the surface of the brain (gray stippled oval in Figure 1B). The drive was mounted inside an inverted 35-mm film canister lid, so that the delicate tetrode connections would be protected by the rest of the canister (black outline) during and between experimental trials. Further details can be found in Siegel et al. (2002) and Hough and Bingman (2004).

While challenging, my colleagues and I have been able to collect high quality data on the representation of space at the neural level in pigeons. The custom-built head-mounted microdrive used three to four bundles of tetrodes created by heat-annealing flexible polyimide-insulated 12-μm nichrome wire (Figure 2; Gray et al., 1995). This allowed the implantation of up to 16 microelectrodes that were resistant to both large- and small-scale animal movements (Siegel et al., 2002; Hough and Bingman, 2004, 2008). This microdrive, secured to the skull using a combination of epoxy-secured stainless-steel screws and dental cement, used a single drive screw to manually advance multiple sets of tetrodes (or separate reference and recording electrode bundles) in 80-μm increments until neurons were isolated on individual tetrode wires. The connector cable from the electrophysiology system to the headstage had a single LEDs mounted on the front (and occasionally on the back as well) that encoded head position, which was timestamped to the neural recordings so we could measure the mean rate of neuronal firing per pixel at a pixel resolution of less than a half-inch square of arena surface. Head direction was calculated either by the difference in X/Y position in two-LED trials or inferred by the change in X/Y pixel position over time in single-LED trials. My own studies added an additional modification; implanting the electrodes at a 30-degree angle toward the midline; this allowed for better sampling of the medial HF subdivisions and avoid the large central sinus blood vessel (Figure 1B, shaded circle) that ran down the midline (Hough and Bingman, 2004, 2008). Using this method, the central sinus was rarely damaged, and the tetrode bundles would remain intact inside a protective inverted film canister for months at a time. These microdrives allowed the study of the relationship between spatial exploration and HF neuron selectivity for several months per research animal.

### Comparisons Between Avian Hippocampal Formation and Mammalian Hippocampus Neural Responses

In a series of studies performed by the Bingman Lab (Siegel et al., 2002, 2005; Hough and Bingman, 2004, 2008; Kahn et al., 2008), approximately two-thirds of isolated, chronically recorded homing pigeon HF neurons were sensitive to particular aspects of the arena. These arenas had different shapes, but generally were enclosed spaces of approximately 6 feet x 6 feet, with walls to prevent escape and a series of pathways that ended in food-containing bowls (either a plus maze in the works by J.J. Siegel, an open arena with local and distal landmarks in the works by M.C. Kahn, or an 8-armed radial maze in the works by G.E. Hough). To encourage exploration, birds were food-deprived prior to experimentation, and encouraged to explore the arena with pellets of food that could be continually replaced *via* outside tubes once birds had visited and left an arm of the maze. And each experiment had a pre-arena period where cells were isolated, a run period of free exploration, and a post-arena period where the animal was returned to a holding area. These methods allowed a consistent environment in which to study the responses of single neurons to various aspects of the exploratory process.

The spatial selectivity of these neurons shared some qualities with other documented response profiles in rodents and primates (location/place and head direction responses) but demonstrated some unique characteristics as well (paths between goals, task context, and pattern responses). In addition, the neural firing characteristics of homing pigeon HF neurons shared some similarities with rodent Hp neurons when analyzed using information theory qualities, but differed in others (see Skaggs et al., 1993 for more detail on these characteristics). These differences and similarities may simply due to the evolutionary divergence of the two groups of animals and the tissues that gave arise to the mammalian and avian structures, but also were likely shaped by the reliance on different spatial navigation strategies. The spatial profiles of avian HF neurons can be grouped into five broad categories (four in pigeons, one in other birds) based on the available research.

**FIGURE 3:**
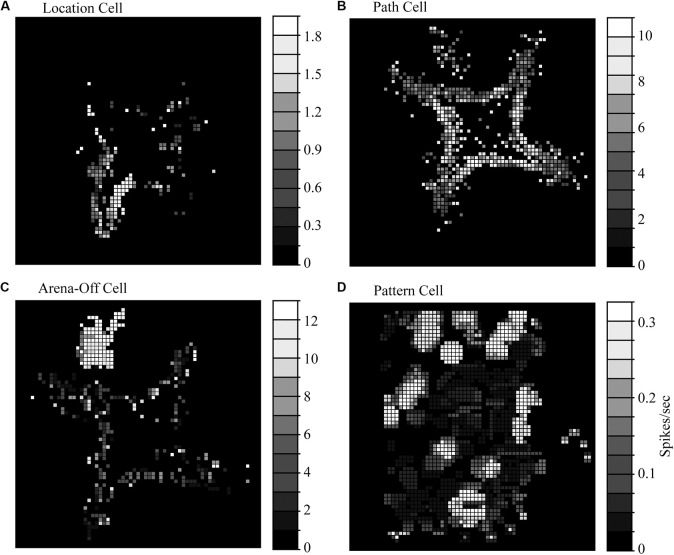
Representative response profiles seen in the homing pigeon hippocampal formation. **(A)** Path cells have a characteristic high firing rate when the animal is traversing between goal locations (red boxes) but less response at goal locations (blue boxes). **(B)** Location cells have their highest firing rates when in a localized area near food bowls. The pattern pictured corresponded to a place-like cell. **(C)** Arena-off cells are presumably context-dependent and fire while in the holding area (in this arena, in the NW corner) but have reduced firing while exploring the arena. **(D)** Pattern cells have multiple regions of high firing in multiple areas of an open arena that appear to be clustered around the arena edges. **(A–C)** Adapted from raw data included in Hough and Bingman (2004); **(D)** adapted from Figure 6A in Kahn et al. (2008) with permission from Elsevier.

The first type of spatially selective HF neuron in the pigeon is approximately half of all spatially selective neurons, and are classified as “location cells.” These neurons increased their firing rates when the animal’s head was in one or more localized areas in a behavioral arena (Figure 3A). These responses were relatively stable in location across a 10–20-min recording session. Similar to the results in rodents (i.e., Muller and Kubie, 1987), rotating arena cues (such as the colors of lights illuminating food bowls) causes a similar rotation in the location-specific firing of Hp neurons (Hough and Bingman, 2008). But unlike rat place cells, pigeon HF location cells have much more variable fields of selectivity. There is a wide range of spatial information per spike, a measurement of how well we can predict an animal’s location using neuronal firing rate, from a very low 0.30 (Siegel et al., 2005) to quantities appearing closer to rodent place cells at 0.5 to 1.5 (Hough and Bingman, 2004). Location cells also have relatively low coherence, which is a measure of the compactness and circularity of areas of high activity (0.34–0.56), though this is on par with some rat literature (i.e., Kubie et al., 1990). They also have relatively low reliability (0.12–0.40), which is the likelihood that every visit to a pixel had a similarly high response rate of firing (Siegel et al., 2002, 2005; Hough and Bingman, 2004). Rodent place cells have relatively higher values in both coherence (0.36–0.81) and reliability (0.55 and above), suggesting a more robust and stable response profile (Quirk et al., 1992; Shapiro et al., 1997; Nitz and McNaughton, 1999).

Location fields in homing pigeons also appear to cluster near environmentally relevant locations in the local environment (such as food sources) and are far less numerous in areas between goal locations (Hough and Bingman, 2004, 2008; Siegel et al., 2005; Figure 3). And many location-selective neurons exhibited more than one preferred location (Hough and Bingman, 2004; Siegel et al., 2005). In these experiments, all food locations were visually similar and had relatively homogenous amounts of available food. Perhaps with more significant differences between goal locations, we would have seen a higher selectivity for a particular goal location for each cell.

**FIGURE 4:**
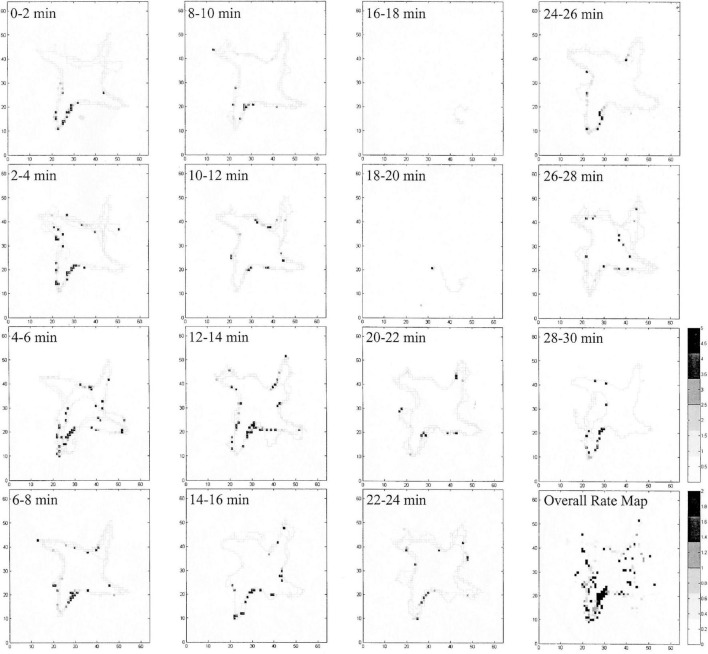
Evidence of a pigeon place cell summarized in Figure 3B, broken into 2-min epochs. The cell consistently and reliably fired while in the entrance to the SW arm of the radial maze (black squares), even though the animal was visiting all four of the baited arms. The holding area portion of the experiment is not included in this figure (in the N area of the map). In two of the epochs, the bird did not move very much (16–18 and 18–20 min), and the cell only fired as it went from the SE to the SW arms.

We occasionally found true place cells in pigeons, which coherently and reliably fired every time the animal passed the same location (Figure 4), but they were rare as being only 7.5% of location cells and were found in only two studies (Hough and Bingman, 2004, 2008). These cells had a high spatial information per spike (0.95–1.79), which is on par with rodent place cells (Nitz and McNaughton, 1999). Admittedly, the number of pigeon place cells may be due to the limited number of recordings made in the dense ventral cell layers of VM and VL, the most likely homolog of mammalian dentate gyrus (Hough and Bingman, 2004, 2008; Siegel et al., 2005), so perhaps pigeon place cells exist in higher quantities than has been detected to date. Place cells have been found in higher quantities in the food-storing tufted titmouse than in the non-storing zebra finch (Payne et al., 2021), which might indicate that place cells are more numerous in avian species that must encode large numbers of specific locations.

The second type of spatially selective HF neuron in pigeons, the “path cell,” is subtly different from other directionally selective neurons in mammalian Hp and was a novel finding at the time (Hough and Bingman, 2004). These neurons were 25% of the spatially selective neurons in the pigeon and had a region of higher firing that connected goal locations (Figure 3B), and perhaps serve a functionally similar role as primate spatial view cells that fire when an animal is looking at a particular location regardless of the animal’s location (Rolls, 1999), or goal cells that fire when oriented toward a goal (Sarel et al., 2017). Path cells significantly increase their firing rates when traveling between goal locations, regardless of the animal’s head direction, velocity, or the distance traveled (Hough and Bingman, 2004). The area of highest firing in these neurons appeared to be long, thin, and curved toward bowls, which may be responding in a manner like goal cells found in bats (Sarel et al., 2017). While some path cells were selective for a single pathway between two locations, most were selective for multiple paths between different locations. In all cases, the responses were not based solely on head direction or velocity, as the cells fired at multiple compass directions and at a variety of animal speeds.

Path cells also appear to be highly sensitive to disruptions between local and distant cues. In a light-rotation experiment, we rotated the colors of lights illuminating food bowls without changing or rotating any other aspects of the arena. While location cells rotated their response fields to match the changes in color, path cell responses broke down into location-sensitive responses, suggesting that path responses were processing more globally relevant aspects of the task (Hough and Bingman, 2008). They also may reflect a greater flexibility in encoding the spatial relationships of objects than present in rodent head-direction cells (i.e., Bingman et al., 2003). Path cells may also have evolved to coordinate the overall progress toward a goal location while in flight, independent from the characteristic proprioceptive feedback gained from walking in a specific direction (such as the goal cells of bats; Sarel et al., 2017).

The third type of spatially selective responses found in pigeons reveals one of the more interesting findings in homing pigeons; the presence of context-dependent responses in the hippocampus (Figure 3C). These neurons had high rates of firing prior to running in the arena (and possibly anticipation of reward), but significantly slowed down their rates of firing once active in the arena (Hough and Bingman, 2004; Siegel et al., 2005). This third type of spatially selective neuron was labeled the “arena-off cell” and comprises approximately 25% of spatially selective neurons. The change in firing didn’t appear to be a loss of isolation- the neuronal profiles (used for cluster cutting out units from background by sampling various action potential shape parameters) did not change even as the rate of firing while exploring the arena decreased to at least a third of the holding area rate. Context-dependent responses have been documented in brown-headed cowbirds (*Molothrus bonariensis*), which suggests that context is an important aspect of spatial navigation (Sherry et al., 2017). While the extent and parameters of context-dependent responses are unknown at present, they might be responsible for rehearsing a task prior to performing it, or perhaps reflect the motivational state of the animal (hungry vs. satiation).

A fourth cell type, “pattern cells,” has multiple patches of higher activity, apparently clustered around the periphery of an open-field arena (Figure 3D). These cells were found in DM and Tr on both sides of the homing pigeon HF (Kahn et al., 2008), and may suggest a fusion of several well-known mammalian types. These types are grid cells, which fire in a distance-dependent pattern across an arena, and boundary cells, responding when the animal is at the edge of an environmental space (Solstad et al., 2008). There are a multitude of reasons why this response category was likely not noted in earlier studies (Siegel et al., 2002, 2005; Hough and Bingman, 2004, 2008). First, the Tr was typically not a subdivision that received a lot of recordings due to the relatively sparse neuronal density, and the tendency to have low firing rates with no obvious patterns; it’s possible they were recorded but not counted as “spatially modulated” due to their inherent noisiness. Another is that researchers were simply not looking for these pattern-like neuronal profiles (grid cells in rodents were not documented until 2005). So, what is the purpose of pattern cells? An intriguing possibility for why pigeons have pattern cells is that they may be a hybrid of grid and boundary cell, responsible for tracking relative distances between landmarks in an open arena where there are no clear pathways to follow.

A potentially fifth type, the “head-direction” cell, is documented in both rats (Rolls, 1999) as well as several species of birds outside of homing pigeons. Streaked shearwater demonstrate strong compass-dependent responses predominantly to magnetic north, suggesting that migration is a salient cue for navigation in that species (Takahashi et al., 2022). In another recent study performed in the Japanese quail, head-directional tuning was found in about 12% of HF neurons, with preferred directions spanning all compass directions (Ben-Yisahay et al., 2021). It is possible this type of response is found in the homing pigeon as well, as compass navigation in pigeons requires a functional hippocampus (i.e., Bingman and Jones, 1994), but has not been documented to date.

Laterality appears to be a common feature of the avian HF when coordinating global vs. local aspects of spatial behaviors, but the exact pattern of left vs. right functionality is conflicting. In an early study, pigeons were only able to use global cues to locate food-containing locations (such as cues on the walls) when the right HF was intact, while animals could use the differences in local cues such as unique objects between food bowls to find the same locations when the left HF was intact (Kahn and Bingman, 2004). In a follow-up neurophysiological experiment, these researchers found less spatial selectivity in left HF neurons compared to right HF neurons; the right HF neurons had fewer arena locations with higher activity, but those neurons exhibited higher coherence and information per spike, and path-type responses were seen more frequently in the left HF (Kahn et al., 2008). In chickens, the global (right HF) and local cue-relevant (left HF) roles appear to be reversed from what is found in pigeons (Tommasi and Vallortigara, 2001; Tommasi et al., 2003) when tested using visual and ablation methods, and more closely matches the laterality pattern seen in humans (Brederoo et al., 2020; Gerlach and Poirel, 2020). While outside the scope of this review, several excellent reviews discuss this and other aspects of lateralization in the avian brain (Rogers, 2008, 2011; Morandi-Raikova and Mayer, 2021).

### Hippocampus Subdivisions Contain Multiple Response Profiles

When mapping out the spatial response profiles, several patterns emerged. For this summary, I am using the framework of Atoji and Wild (2006) to delineate subdivisions. First, the entorhinal-like DL area contained neurons that were location-sensitive and those whose patterns lacked easy identification, in approximately equal percentages. The DM subdivision contained a mixture of all responses (location, path, and arena-off) in equal quantities. The VL/VM region (their close anatomical proximity prevented easy separation into specific subdivisions) contained most of the path cells, and a few location cells. The three pigeon place cells were likely in the VM subdivision due to their being recorded just prior to electrodes entering the void between the hemispheres; this subdivision has been suggested to be the brain area most comparable to the dentate gyrus of mammals (Herold et al., 2015; Atoji et al., 2016). Pattern cells are found in both hemispheres in Tr, and perhaps DM as well (Kahn et al., 2008). Therefore, when attempting to map out response profiles to subdivisions of the avian HF, while patterns have emerged with respect to localizing specific aspects of a spatial task to subdivisions, there is also significant overlap of subdivisions and response types. This uncertainty as to how the HF operates as a coherent whole to process spatial cues would be best answered by researchers using more modern techniques and multi-array recordings.

## Discussion

The differences between avian and mammalian response profiles suggest that adaptive specialization has pushed the avian HF to develop responses that fit the navigational style of flying animals. There appear to be at least five broad categories of responses in avian HF neurons, four of which are found in homing pigeons: location cells that encode an animal’s position, path cells that coordinate travel between goal locations, arena-sensitive cells that encode context, and pattern cells that might encode distance traveled. A fifth type found in other birds, the head-direction cell, might be present but has not been documented in the pigeon. A bird in flight needs to know where it is, where it wants to go, what it needs, and how to track its progress to a goal using the flow of visual information it receives while in transit in the absence of proprioceptive feedback. By studying these types of responses in more depth, and with a larger sampling of both sides of the hippocampus at one time, and more samples for each subdivision, we can get a clearer picture of how selective pressures have created a brain area that is fundamentally similar to mammalian Hp using a much different area of the brain. For example, it would be interesting to see if there is a difference in the relative proportion of path cells and head-direction cells in species that are using the HF to remember salient locations in the environment such as scatter hoarding birds or homers (path > head direction) vs. compass orientation to coordinate seasonal migration (head direction > path). Another question is whether these responses differ in terrestrial birds vs. flying species; does the primary locomotive manner correlate with the relative proportion of these responses in the HF across birds and mammals?

Since the majority of homing pigeon HF electrophysiology studies were performed over a decade ago in a handful of studies, there are more questions than answers. First, we do not know if the areas of higher responsiveness in the various response categories are stable across days in the same experimental space, or whether changing the context of cue locations (such as baiting different locations across different recording sessions) changes the response profiles of individual neurons. While most isolated HF neurons can be successfully recorded for over an hour in a single recording session, the act of removing a bird from the tether and reattaching it on a subsequent day tends to move the electrodes just enough to prevent re-isolation of the same cells. It is possible that current wireless technology would allow for the repeated recording of isolated neurons across sessions without losing them to the headstage movement that occurs when handling animals to attach the recording cable.

Another question is whether the variability in HF neuroanatomy that occurs both seasonally and throughout the lifespan changes the spatial response profiles. The seasonal variability in HF volume and density is well documented in food-storing passerines (Smulders et al., 1995, 2000; Sherry and Hoshooley, 2010). In particular, the avian HF tends to get larger in warmer months when food storage occurs, and smaller in cooler months when retrieval happens (Sherry and Hoshooley, 2010), and increases in HF complexity have been correlated to increases in spatial learning capabilities (Pravosudov and Clayton, 2002; Hoshooley et al., 2007; Hoshooley and Sherry, 2007). When new neurons are added to or removed from the HF, there is likely a concomitant change in HF neuron spatial profiles as well, even though birds with higher spatial memory demands may not always have significantly larger hippocampal volumes or neural densities (Pravosudov et al., 2002; Kozlovsky et al., 2017, but see Volman et al., 1997 where this trend does appear in woodpeckers). Another change is age-related; the hippocampal system appears to get more dense but less selective as animals age (Coppola et al., 2015, 2016), when there is a significant decline in spatial working memory in pigeons (Coppola et al., 2014a). It would be intriguing to see how the different types of spatially selective neurons change their response profiles throughout the lifespan, and how these changes underlie the decreased memory performance seen in older birds.

Another limitation in prior studies has been the use of tethered animals in small, enclosed arenas. Therefore, we do not know how the avian HF encodes large-scale spatial characteristics (such as during homing or migration) at the neural level, or how spatially sensitive neurons respond to changes in non-visual cues like magnetic fields or scents, two complimentary senses that pigeons use to navigate (Vargas et al., 2006; Gagliardo et al., 2009). Now that wireless technology has advanced to allow for neuronal recording from untethered animals (Sarel et al., 2017; Nourizonoz et al., 2020), future experiments should be performed to see how the avian HF encodes large-scale three-dimensional spatial information during flight. We do know that bats appear to have spherical place fields while flying (Duncan and Henson, 1994; Yartsev and Ulanovsky, 2013; Dotson and Yartsev, 2021).

In conclusion, homing pigeons are an attractive novel organism for understanding how spatial memory demands can influence the representation of space at the neural level. Using the advancements in neurophysiological methods over the last decade, it will be interesting to see how new researchers and methods further investigate the neural bases of navigation in homing pigeons. The combination of electrophysiology and homing experiments will provide us with new avenues of research in understanding how the hippocampus encodes environmental space.

## Author Contributions

The author confirms being the sole contributor of this work and has approved it for publication.

## Conflict of Interest

The author declares that the research was conducted in the absence of any commercial or financial relationships that could be construed as a potential conflict of interest.

## Publisher’s Note

All claims expressed in this article are solely those of the authors and do not necessarily represent those of their affiliated organizations, or those of the publisher, the editors and the reviewers. Any product that may be evaluated in this article, or claim that may be made by its manufacturer, is not guaranteed or endorsed by the publisher.
